# Protection of Privacy of Information Rights among Young Adults with Developmental Disabilities

**DOI:** 10.1007/s11469-018-9904-x

**Published:** 2018-04-18

**Authors:** Nazilla Khanlou, Anne Mantini, Attia Khan, Katie Degendorfer, Masood Zangeneh

**Affiliations:** 1 York University, 4700 Keele St., Toronto, ON M3J 1P3 Canada; 2 Toronto, Canada; 3 University of Guelph-Humber, 207 Humber College Blvd, Etobicoke, ON M9W 5L7 Canada

**Keywords:** Information privacy rights, Canada, PIPEDA, PHIPA, Confidentiality, Personal information protection

## Abstract

Protection of privacy of information for young adults with developmental disabilities and their families is essential to promote quality of life, well-being, empowerment, and inclusion. Despite this, the young adults’ information privacy rights are increasingly at risk. This paper provides a scoping review, applying Arksey and O’Malley’s (2005) approach, of all published peer-reviewed journal articles and gray literature to examine the barriers and facilitators in utilization of legislation that protects the collection, use, disclosure, and access of personal information in Canada. The scoping review process was further expanded with a rigorous reliability method and applied a socio-ecological framework to the final 47 studies. National and international policy and legislation (macro level), organization-based factors (meso), young adults and community interactions (exo), and individual disability related factors (micro) are examined. The review identifies the barriers and highlights the importance of facilitators for acting on personal privacy rights.

Young adults with developmental disabilities (YADD) and their families are at risk of exploitation in terms of privacy and protection of their personal information (Joffe, 2010). Their increasing dependency on multiple community-based and private agencies creates a scenario where a wide range of personal information is retained and shared through the course of servicing these individuals and their families over time. YADD are vulnerable not only because they are in a sensitive period transitioning into adulthood but also as they have complex developmental needs and may experience difficulties making their own decisions (Dyke et al., 2016). They may also have concurrent health and mental health needs which require support, and even as adults may frequently depend on secondary decision makers to protect their privacy of information rights (Geist, 2016).

Developmental disabilities (DDs), which include intellectual disability, autism, down syndrome, fragile x syndrome, cerebral palsy, and developmental delays, are lifelong and affect multiple aspects of psychosocial development, physical functioning, and participation in daily activities (Developmental Services Ontario (DSO), 2016; Roebuck et al., 2008). Individuals with DDs may have deficits in their cognitive, decision-making, comprehension, and communication capacity (Roebuck et al., 2008) that increases their exposure to human rights violations related to privacy of information (Fogden et al., 2016). They or their family caregivers and service providers often do not have the necessary resources to defend their privacy rights to personal information.

While legislation in Canada is well developed and in place to guide the protection of personal information (e.g., Personal Information Protection and Electronic Documents Act (PIPEDA), 2000), and health information for individuals (e.g., Personal Health Information Protection Act (PHIPA), 2004), the translation of relevant legislation as required for individuals at-risk of understanding or communicating their rights is not. This impression is informed by our findings from our recent and on-going research studies on families of children and young adults with developmental disabilities (Khanlou et al., 2017a; Khanlou et al., 2017c; Khanlou, 2018). During the data collection phase of these projects, family caregivers, service providers, and YADD frequently reported concerns they faced with the collection and use of their personal information. Specifically, they relayed concerns about the extensive paper work, dispersed services, navigation across multiple service sectors with repetition of requests for personal information, financial difficulties, social isolation, and stigma. Immigrant family caregivers of YADD faced additional information privacy concerns specific to language fluency, lack of social networks, and new knowledge required post-migration of service delivery systems and their rights, which were reported to affect their access to and utilization of health and social services (Khanlou et al., 2017a; Khanlou et al., 2017b; Khanlou et al., 2017c; Khanlou, 2018). Their concerns and these factors influence the privacy rights of YADD in a challenging manner. Many YADD and their parents continue to remain uncertain as to who is accountable for the protection of their privacy rights given the complexity and persistence of developmental challenges of the young adults.

In Ontario, Canada, persons with DDs are permitted to remain in their secondary school until they reach 21 years of age. Thus, even though they are adults as of 18 years of age, their privacy and confidentiality rights are largely protected by the adherence to privacy laws within their school environment. However, following completion of school, the young adults with developmental disabilities can take different pathways ranging from partial employment, volunteer work, vocational training, day programs, or to no involvement in activities outside the home. Once these young adults leave the protection of their school settings, they are increasingly dependent on both private and public agencies from within both the social and health sectors. This dependency on multiple organizations to provide needed services, supervision, care, employment, or education— commercially based or not—increases dramatically as the young adults transition into adulthood.

Given the significant gap between application of relevant legislation or information privacy evidence and the needs of young adults with developmental disabilities, the YADD Privacy Project was developed (Khanlou, et al., 2017b). As part of the project, a methodologically rigorous scoping review was conducted to address this gap by synthesizing evidence on information privacy rights for YADD in Canada. The goals of this comprehensive scoping review of both published peer-reviewed articles and online gray literature were to: (1) examine the range, depth and nature of both the scholarly and gray literature related to access to privacy of information rights and utilization of legislation in Canada for the provision of services to young adults with developmental disabilities (PIPEDA, 2000 or PHIPA, 2004), (2) apply a socio-ecological framework (Bronfenbrenner & Ceci, 1994) to the findings to illustrate the barriers and facilitators of information privacy rights for young adults with developmental disabilities, and (3) to contribute to the development of future strategies and solutions enhancing information privacy rights with evidence-based recommendations.

## Methods

Our protocol was developed using the scoping review methodology proposed by Arksey and O’Malley (2005), which has a five-step process for conducting scoping reviews. This method entails (1) identification of the research question, (2) identification of relevant studies, (3) study selection, (4) charting the data, and (5) synthesizing and reporting of the results. In addition, we refined the scoping review process to increase methodological rigor, by including three more components as suggested by Tricco et al. (2016) and Colquhoun et al. (2014) and their colleagues, including establishing a protocol, utilizing at least two reviewers and calculation of reliability, a reporting checklist, and conducting a consultation exercise to ensure our results are useful to advancing the field.

**Fig. 1:**
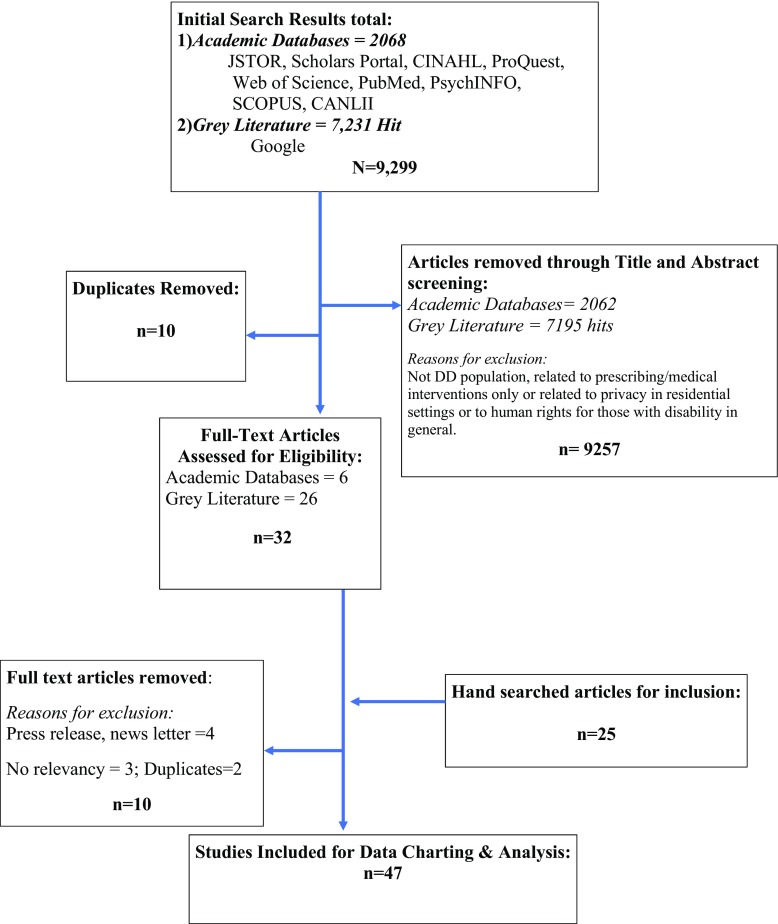
Information privacy rights PRISMA diagram

- Step 1 We identified the research question as follows, “What does the scholarly and grey literature on information privacy in relation to PIPEDA or PHIPA inform us about the barriers and facilitators young adults with developmental disabilities encounter when accessing and utilizing their privacy rights?”
- Step 2 Next, relevant peer-reviewed studies were identified by searching electronic databases: ProQuest, JSTOR, Scholars Portal, EBSCO, Web of Science, PubMed, PsychINFO, Scopus, and CANLII (for legal cases and briefs), using specific search terms: PIPEDA OR PHIPA OR information privacy AND developmental disabilities AND Canada. The gray literature search was conducted using the Google search engine, and keywords/phrases were either (a) barriers to PIPEDA for young adults with developmental disabilities in Canada or (b) barriers to PHIPA for young adults with developmental disabilities in Canada. The database searchers were not limited by language or type of publication, but location for keywords was restricted to Canada. Specifically, we searched Google Search and websites of agencies that service YADD. The electronic searches retrieved 9299 articles (Fig. 1), and 25 articles through hand searching. Ten duplicates were removed. Search and keyword strategy were developed by research team members and approved by the study Principle Investigator and the health sciences librarian.

- Step 3 Abstracts of identified articles were reviewed to assess if they met the inclusion and exclusion criteria. Articles were eligible if they were written in a peer-reviewed journal or published online, including policy documents, websites, and commentary, between January of 2000 and November of 2017. All titles and abstracts were individually examined by the reviewers AM and AK. We excluded any articles or gray literature that was aimed at the general population or cited the legislation without detailed explanation or information on application in servicing YADD. Articles not pertaining to access to or utilization of privacy legislation were excluded. Abstracts were included if they described issues related to PIPEDA or PHIPA and legislation with young adults with developmental disabilities. We included articles based on other populations (e.g. physical disability, and mental illness) and countries (EU) and USA) only if their findings were also relevant to the developmental disability community. Articles discussing children and youth under the age of 16 were not included, and studies of adults were only included if a large portion of the reported sample in the study included young adults under 28 years of age. To ensure comprehensiveness in the evidence base, we also supplemented our list of included articles with academic literature that discussed information privacy with similarly vulnerable populations (e.g., those with mental health or decision-making capacity issues). We included studies conducted outside of Canada when findings were informative for barriers and facilitators of privacy legislation. Forty-seven articles (24 peer-reviewed and 23 gray literature) fulfilled the eligibility criteria for the scoping review.
- Step 4 Information was captured on Excel 2011. To ensure reliability between reviewers, a series of training exercises were conducted and inter-rater agreement for both the study inclusion and data charting phases. Changes were made based on feedback until there was consensus regarding the face validity of the tool by the two reviewers. A total of 10 randomly selected articles were evaluated by two reviewers, and 5 by three reviewers, to assess the level of agreement across all categories for each article. Reliability for level of agreement on items on the abstraction form ranged from 61 to 96%. Given the high degree of variability among the published and gray literature, the mean level of agreement obtained (79.5%) was deemed to be sufficient. All changes were verified by reviewers to ensure data accuracy.

## Results

All analyzed articles described legislation focused on information privacy rights, in relation to collection, use, disclosure or sharing and correction, or access for checking. Eighty percent of the articles (peer-reviewed published and gray literature) also addressed confidentiality and/or autonomy while only 6 articles (12%) reviewed ethical issues related to accountability on the part of the service organization. Full-length manuscripts, case studies, legislative acts, policy reports, professional practice guidelines, service provider resources as well as literature and policy reviews were obtained for all abstracts identified for inclusion by both reviewers.

### Location

Fifty-one percent of the 47 articles included for full text review and synthesis represented peer-reviewed published articles and 48% represented documents selected through the gray literature search. Of the published articles, five were based in the USA and three in the EU.

### Population

While 39 articles were related to young adults with developmental disability, 8 articles from the published and gray literature did not discuss young adults or developmental disability, but to consumers of technology (*n* = 4), genetic researchers (*n* = 1), and patients with mental health diagnoses (*n* = 3).

### Legal and Ethical Themes

Within the whole sample (*n* = 47), all articles discussed relevant legal and ethical issues but through a variety of sub-themes. Fifty-five percent of articles discussed collection of personal information, while 42 to 45% focused on use disclosure of personal information and 25% discussed the legal and ethical issues related to correction or access to personal information that is kept within the organization. With respect to ethical principles related to the privacy of information rights, 83% focused on confidentiality, 38% on the need for autonomy, and 13% discussed accountability on the part of the organization collecting the personal information.

### Types of Articles

Articles from both the peer-reviewed published and gray literature included a variety of formats, from commentary to policy/legislation and literature reviews to empirical studies utilizing qualitative and/or quantitative data, and professional or organization-based guidelines. Policy and legislation reviews or discussions accounted for 22% of the total articles, while 17% represented the empirical research studies and 32% were based on reviews of the literature (please see Table 1). Fifty-six percent of articles representing the gray literature were published as guidelines, either to be used as a general resource to consumers of the organization (*n* = 15), or to support health professional practice (*n* = 5), or to communicate the organization’s policy and procedures (*n* = 6). Of the five articles published as professional practice guidelines specifically addressing privacy rights for use by health professionals (e.g., Occupational Therapists, Speech/Language Pathologists, Personal Support Workers), none discussed relevant parts of the legislation, or potential strategies to help the professional protect information privacy rights of those they are supporting. Rather, they referred the reader to read the PIPEDA. The remaining 46% of published gray literature included websites, information sheets, press releases, reports to the Canadian parliament, dissertation, relevant legislation, and a document of meeting minutes (please see Table 2).

**Table 1:** Studies on privacy of personal information: study characteristics (*N* = 24)

	Author	Type of study	Participants	Country and law	Type of information	Privacy rights addressed	Ethical principle addressed	Barriers identified	Facilitators identified
1.	Mills et al., 2003	Policy analysis	Not applicable	Canada: PIPEDA PHIPA	Health information	Collection Disclosure	Not addressed	1. Lack of shared databases 2. Poor integration Of policy and technology 3. Lack of agreement on keeping privacy and security of PHI 4. Lack of commitment to technological networks	1.Increasing use of “anonymising” data through encryption 2. Advocating for patient privacy 3. Establishing strong policies 4. Willingness to invest in technological advances
2.	Lafky & Horan, 2011	Mixed methods: qualitative interviews and quantitative survey	PHR users: unwell, disabled and well adults	USA	Personal health information	Use Access	Confidentiality (breach) Autonomy	1. Low levels of experience with managing PHI 2. Disabled less concerned, and less in control with privacy 3. Lower engagement in privacy-protecting activities 4. Underrepresentation of disabled people 5. Records which may be scattered among multiple providers	1. Heightened access and sense/perception of control for those that are more concerned with privacy and more able to protect their health records privacy
3.	Dyke, et al., 2016	Comparative legal and policy analysis	None (qualified researchers)	Canada PIPEDA	Health information	Disclosure	Confidentiality	1. Not complying with PIPEDA legislative requirements 2. Difficulties identifying jurisdiction 3. Complicated legal framework for privacy law (public and private; federal and provincial) 4. Effort and cost of sharing, protecting, and ability to publishing 5. Lack of coherence between national and international privacy frameworks 6. Lack of trans-border data flow sharing	1. Understanding local norms and standards and legal requirements 2. Understanding factors influencing consumer adoption of health information management tools 3. Using an accountability model for ensuring that Canadian privacy standards are in place
4.	Yalon-Chanimiz, 2009	Review of literature and a conceptual model	Adults with intellectual disability	USA	Personal health information	Collection access	Autonomy	1. People with ID experience processing delay, low literacy, stigma 2. Complexities in accessibility: verbal communication, layout of physical environment, product-operating instructions and procedures	1. Provision of: extra time, age appropriate information, simple language, simple layout, pictograms, and auditory means 2. Higher levels of self-determination: more use of services, inclusion in their community, reducing stigma 3. Training and education for service providers
5.	Urowitz et al., 2008	QUAN (National Scan)-survey - prospective, cross-sectional	CEOs of hospitals (*N* = 83)	Canada PHIPA	Electronic health records	Use and access	Confidentiality	1. Absence of technology to facilitate the electronic health record 2. Financial resources 3. Patient computer literacy 4. Lack of hospital survey on patient needs for HER 5. Unwillingness of hospitals to providing patients with access to their EHR	1. Use of electronic patient portals 2. Cultural shift of healthcare providers: to give up “ownership” of the files
6.	Siegel, et al., 2009	Comparative legal and policy analysis	Consumers of technology	Canada PIPEDA USA, EU	Personal information	Use (security breach)	Accountability	1. Exploding growth of social media, yet lacks detail how information is used for advertising, and how to verify consent from non-users 2. Regulations are “piece-meal” not explained enough 3. Developments in Canada have been only advisory	1. Jurisdiction over foreign organizations 2. Clear, consistent communication as to the use of the information across platforms
7.	Geist, 2016	Critical review of legislature	Consumers of technology	Canada PIPEDA	Personal information	Disclosure (security breach)	Confidentiality accountability	1. Corporate, lobby group focus on commercial concerns 2. Absence of “order-making power” 3. Lack of penalties for privacy violations 4. Weak protections when outsourcing information to low protection jurisdictions	1. Public engagement, protests for privacy rights 2. Mandatory breach disclosure requirements
8.	Larivie’re-Bastien, & Racine, 2011	Review	Adolescents with cerebral palsy	USA	Health information	Collection	Confidentiality autonomy	1. Failure to adjust and focus on disability prevents focus on traditional adolescence issues 2. Lack of preparation to the transition and social isolation 3. Difficulty obtaining health information 4. Needs for confidentiality and privacy are not respected and not acknowledged 5. Issues of informed consent and capacity often not addressed: opportunity to take part in decision-making not provided enough	1. Positive attitudes and trustful, open provider-patient relationships free of stereotypes 2. Technology for communication, time and resources help with autonomous consent 3. Healthcare providers can facilitate implicit personal autonomy even if it contradicts professional opinions
9.	Austin, 2006	Review: case and legislature	Not applicable	None identified	Consumer information	Collection use	Confidentiality autonomy	None identified	1. Privacy protection shifts balance of power away from business to consumers 2. Specifics of privacy give consumers clear ability to control privacy 3. Enforcing organization to comply with explicit consent for sensitive information
10.	Repetto, et al., 2008	Review	1. YADD in transition: from Grade 6 to 12 students 2. Family caregiver 3. Teachers	USA HIPPAA FERPA	Personal information	Collection use disclosure Access	Confidentiality Autonomy	1. Legislature limited to record keeping and documentation and may not cover the broader needs 2. Young adults with DD do not have the knowledge, skills and are not health literate 3. Standards, curriculum materials and legislation do not refer to disabilities 4. Insufficient expansion of transition planning 5. Cannot reveal students’ personal health information as they learn, so makes teaching the content difficult.	1. Improved guidelines for teachers to support confidentiality within the classroom 2. Teaching students how to balance the advantages and disadvantages of releasing personal health information 3. Empowering and preparing students to make informed choices related to disclosure and increasing their communication, reasoning and investigating skills for health promotion
11.	Chan, & O’Brien, 2011	Review	Not applicable	Canada PHIPA	Health information	Use	Confidentiality Autonomy Accountability	1. Precarious balance between respecting client confidentiality and facilitating patient care 2. Caregivers who assume an allied care giving role not recognized as health information custodian	None
12.	Gagnon, et al., 2016	Qualitative study	Canadian service providers	Not identified	Electronic personal health records	Use Access Disclosure	Confidentiality Autonomy	1. Confusion, lack of awareness 2. Usability and relevance with system design 3. Weak user capacities and attitudes (i.e., patient health literacy, education and interest, support for professionals) 4. Environmental factors (i.e., government commitment, and targeted populations)	1. Increased guidance for supporting staff and for defining ePHR, data ownership 2. Access to information and sharing of health records to inform stakeholders
13.	Joffe, 2010	Policy review	Applied for people for DD	Ontario Human Rights Commission Code	Health information	Collection	Confidentiality Autonomy	1. Lack of knowledge 2. Confusion- > which rights apply in which contexts 3. Complaints procedure: not enough support, and fear of reprisal from service providers	1. Accessible and understandable information 2. Helping people to develop as self-advocates 3. Rights education outside of service provision: ensures confidentiality 4. Provision of accommodations and supports to participate in Act 5. family member/friend support the person with disabilities
14	Keith, 2004	Commentary	Not applicable	Canadian PIPEDA and USA privacy legislature	Personal information	Collection Use Access	Confidentiality	1. Unclear language 2. Lack of direction in Act 3. Stringent definition of personal information	1.Clarity of purpose in collecting personal information 2.Consent 3. Limiting collection and use 4. Open to public review and compliance with requests for personal information access
15.	Rule, 2004	Policy review	Not applicable	PIPEDA	Personal information	Collection Disclosure	Confidentiality	1. Lack of distinction between “strategic and consummatory privacy rights 2. Unknown or undefined risks 3. Logic of markets vs. logic of personal rights 4. Manipulation of personal information connected to online transactions 5. Risks: profiling, matching, data mining	1. Clarify purposes for gathering and withholding information 2. Clarify how information is shared
16.	Beardwood, 2015	Review	Not applicable	PHIPA and PIPEDA, Canadian Digital Privacy Act USA EU	Personal health information	Disclosure (privacy breach)	Confidentiality Accountability	Not identified	1. Organizations knowingly contravening the Reporting Obligation or the Notification Obligation are guilty of punishable offense 2. Nothing unique or systemic required to report a privacy breach to Commissioner.
17.	Clement, & Obar, 2016	Review	Canadian consumers of internet	PIPEDA	Personal data of customers	Use Disclosure	Confidentiality	1. Each company report is idiosyncratic-hard to compare/understand company’s statistics 2. Lack of transparency: retention periods for personal information, physical location of servers, data storage, and facilities where personal information is routed	1. Transparency reporting sheds useful light on previously hidden practices
18.	Davidson, et al., 2016	Comparative review of international legal frameworks	People with mental health problems	PHIPA	Health information	Collection	Autonomy	1 Laws based on mental disorder and risk, rather than decision-making ability 2 Complex overlaps and some logical inconsistencies of guardianship and other mental capacity laws discriminate against people with mental health problems	Universal shifts from institutional to community-based care
19.	Peekhaus, 2008	National survey	General public	PIPEDA, PHIPA, and other provincial Health Information Acts	Health information and genetic data	Collection Use	Confidentiality Autonomy	1. Commercial exploitation of personal information 2. Domestic and international pressures for minimum standards of protection for personal information 3. Ministry of Health use of identifiable health information may not sit well with the Canadian public 4. Compounded safeguarding the privacy of medical information: as medical treatment migrates to complementary and alternative medicine	Willingness of Canadians (61%) to sharing their genetic information with family members
20.	Rose, & Rose, 2014	Review of privacy law	Consumers of healthcare	PIPEDA, PIPA in Alberta and BC, Quebec Privacy Act, PHI, HIPAA and HITECH	Personal health information	Disclosure	Confidentiality	None identified	None identified
21.	Seelig, 2006	Case study	Youth (24 years) with Angelman Syndrome	HIPAA	Personal and health information	Access	Confidentiality	None identified	None identified
22.	Skouge, et al., 2007	Description a model	Young adult in transition	None identified	Personal health information	Collection	Confidentiality Autonomy	None identified	None identified
23.	Moore, et al., 2016	Full research report; mixed methods study	Primary healthcare users in Southeastern Ontario	PIPEDA	Personal health information	Collection	Confidentiality	None identified	None identified
24.	Wolbring, & Leopatra, 2013	Cross-sectional study	Staff of disability service organization	PIPEDA	Electronic health records	Collection Use	Confidentiality Autonomy	1. Very limited control over the collection and safe keeping of personal information created over the course of participants lives 2. PI moderated through staff members	1. Staff more skeptical towards certain sensor applications than others 2. Staff concerned over moderate control of own privacy and their client’s even lesser control over their privacy

**Table 2:** Gray literature on privacy of personal information: study characteristics (*N* = 23)

	Organization or author	Type of document	Population of focus	Country and law	Type of information	Privacy law addressed	Ethics addressed	Barriers identified	Facilitators identified
1.	Central East LHIN, 2009	Final report-guidelines	Young adults in transitional age	Canada: PHIPA	Health information	Collection	Confidentiality Autonomy	Developmental disability identified as a barrier	1. Client-centerd care 2. Respect and avoidance of labels that stigmatize
2.	Shimmell, & Gioia Di Vincenzo, 2017	Professional guidelines: student guide	Occupational therapy students in placements	Canada: PIPEDA PHIPA	Personal information	Reference to Act	Confidentiality	None identified	None identified
3.	Desai, 2013	Final report\-community mental health strategy and policy review	Service providers and criminalized person with mental health problems	Canada: PIPEDA PHIPA	Health information	Use Disclosure Access	Autonomy	1. Families not considered as partner in care 2. Hesitancy to release client’s health information from agency files 3. No centralized information delivery model 4. Not enough consent policies for vulnerable populations with mental health issues	1. Stricter rules on ”circle of care” where consent is not required 2. Understanding the type of information that can be released by whom and how
4.	Canadian Association for Community Living, 2011	Policy review	Young adults with ID	CACL’s Vision 2020	Health information	Collection	Autonomy	1. Attaining full citizenship in law, policy, and practice 2. Institutional approach-insufficient access to educational aids/devices 3. Prejudice, poverty, lack of employment equality, lack of economic security for families 4. Lack of data collection on young people with DD	1. Removal of stigma 2. Specialized services 3. New policies to regulate restraint use in residential services, and for reporting of violence/abuse against those with ID
5.	Community Living- Huntsville, 2012	Not applicable	Not applicable	Not identified	Personal information	Use	Confidentiality	None identified	1. Stressing protection of privacy and confidentiality of personal information 2. Providing supervision in least intrusive manner, with respect for person’s right to privacy and dignity and ensuring safety and well-being
6.	Ontario LAW: Services and Supports to Promote the Social Inclusion of Persons with Developmental Disabilities Act, 2008	Legislation review:	Person with DD/ID	PIPEDA Disability Supports Act	Personal information	Collection Use Disclosure	Confidentiality	None identified	1. Limits on collection and use to no more than is reasonably necessary to meet the purpose
7.	Law Commission of Ontario: Background and Contexts in Which the Law Operates, 2017a	Policy review-chapter	Not applicable	PIPEDA	Information held in capacity registry	Collection Use Disclosure	Confidentiality Autonomy Accountability	1. Complying with privacy protections precludes persons from using informal supports and arrangements and makes it difficult for family members to obtain or share information 2. Checks and audits, and capacity assessment intrudes on privacy	1. Formal substitute decision-making arrangements will give families greater access to supports or ease the difficulties of providing care 2. Use of informal network to assist decision-making
8.	Law Commission of Ontario: Legal Capacity, Decision-making and Guardianship: Final Report, 2017b	Policy/legislation review and qualitative study	Not applicable	Health Care Consent Act, Substitute Decisions Act, Mental Health Act,	Personal information	Collection	Confidentiality Autonomy	1. Vagueness in law 2. Formal assessment of capacity perceived as an invasion of privacy 3. Default role to family-not always involved	1. Greater involvement of families 2. Informal social networks to reduce intrusion on privacy 3. Supporters appointed under decision-making capacity rules required to maintain confidentiality of information
9.	Mental Health Commission of Canada, 2015	Review of policy and literature	Emerging adults 16–25 years with mental health problems	PHIPA	Health information	Collection	Confidentiality	1. Clinicians strictly adhering to altered legal status at 18 for confidentiality requirements 2. Treatment compliance is reduced	Clinicians being “family-engaged”
10.	Kindred Home Care, 2012	Resource	Personal support workers	PIPEDA PHIPA	Health information	Use	Confidentiality	None identified	None identified
11.	Simcoe York Dual Diagnosis Education committee, 2015	Resource	Families with persons with ID + mental health problems	PHIPA	Health information	Collection	Confidentiality	Lack of information regarding services/supports	1. Specific lists of supports and services 2. Specific questions to ask and directions to follow
12.	Wappel, 2007	Policy review	Not applicable	PIPEDA PHIPA	Personal information	Access (have information corrected)	None identified	1. Lack of definition for ”work product” 2. Confusion and lack of specificity between expressed, implied, and opt-out	Clear definition of personal information
13.	Wedge, 2014	Dissertation	Older persons	PIPEDA	Personal information	Collection	Accountability	1. No means for privacy for those who suspect crime against older person to report	None identified
14.	Wellington North, 2015	Meeting minutes	Not applicable	PIPEDA MFIPPA	Health information	Disclosure	Confidentiality	1. Social media use 2. Lack of knowledge of PIPEDA	1. Identify specific strategies for invasion of privacy 2. Only release health information according to legislation
15.	Community Living- Central Huron, 2015	Policy document	Agency’s board of directors, staff, members-at-large and the people receiving service, and those with DD	PIPEDA	Personal information	Disclosure	Confidentiality	Not identified	None identified
16.	Office of the Privacy Commissioner, 2016 Annual Report to Parliament on the PIPEDA and the Privacy Act, 2015–2016	Report to the Parliament	Genetic testing users	PIPEDA	Personal information	Collection Use Disclosure (breach of information)	Confidentiality	1. More information is collected: less adequate safeguards, increased risk and potential consequences of privacy breaches (related to genetic testing) 2. Cross border privacy issues increasing	Breach reports to OPC growing every year, (more since 2014)
17.	Kitchener Downtown Community Health Centre, 2012	Client related policy document	All users of primary care, treatments, referrals, health promotion	PHIPA	Personal information	Collection Use Disclosure	Confidentiality	1. Court order or subpeona	1. Training staff to effectively communicate with persons with disabilities. 2. Clarifying the collection, use and disclosure of personal health information
18.	Legislative Assembly of Ontario: Review of the Personal Health Information Protection Act, 2008	Committee documents/review	People including those with disability	PHIPA	Health information	Disclosure Access	Confidentiality	1. Definition of “health information custodian” not entirely clear 2. Inability to access their own records due to exorbitant fees leads to people with disabilities being denied social assistance, insurance, and accommodations	1. Right to education for patients under the legislation (consent, breaches of privacy) 2. Education for stakeholders to ensure they are aware of their responsibilities under the legislation.
19.	Ministry of Health and Long-Term Care (MOHLTC), 2006	Service policy manual	Persons recovering from treatment, and elderly persons	PHIPA Health Care Consent Act (HCCA), Substitute Decisions Act (SDA)	Health information	Disclosure Access	Confidentiality Autonomy	1. Provision of care under CCAC is subject to sharing of health information	None identified
20.	Renfrew County Catholic District School Board, 2014	Policy and procedure document	Service providers	PHIPA	Personal and health Information	Collection Use	None identified	None identified	None identified
21.	Trottier & Kaattari, 2010	Resource guide	Ontario’s community literacy agencies on finances, administration and employment	PIPEDA	Personal information	Collection Use Disclosure	Confidentiality	None identified	None identified
22.	Lacobucci, 2014	Independent review	People in crisis	PHIPA	Individual’s healthcare information	Collection Use Disclosure	Confidentiality	1. Constraints on information-sharing issues makes coordination between the TPS and the mental health system less effective	2. Healthcare institutions must protect physician-patient confidentiality and sharing of healthcare information.
23.	Luker, 2009	News letter: College of Audiologists and Speech-Language Pathologists of Ontario	Persons with, hearing, speech, and language difficulties	PHIPA	Health information	Access (correction of information)	Confidentiality	None identified	None identified

## Discussion

**Fig. 2:**
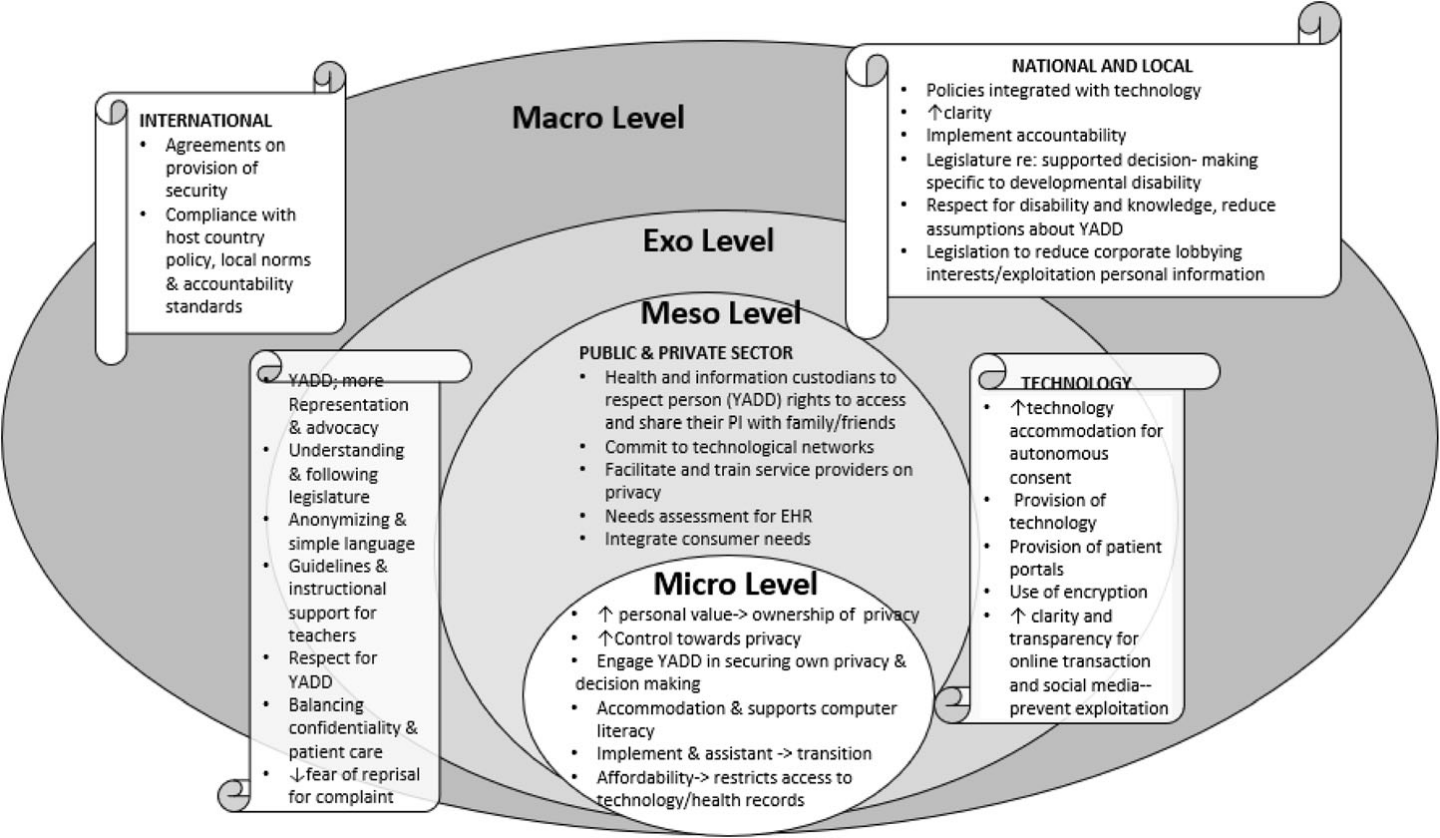
Conceptual framework: determinants of privacy of personal information rights for YADD

Despite legislation that provides standards on how to collect, use, and disseminate personal information to protect privacy, this review identifies specific factors as barriers and facilitators to access and use of information privacy rights for YADD and their families. These findings were classified according to Bronfenbrenner’s socio-ecological framework (Bronfenbrenner & Ceci, 1994) of classifying macro level (policy, legislation, attitudes), meso level (organization), exo level (community interactions), and micro level (individual, family), to more fully understand influences on access and utilization of privacy rights for young adults with developmental disabilities (please see Fig. 2).

### Macro Level: Policy and Legislation (National and International)

At the macro level, this review suggests that aspects of policy and legislation, both in Canada and internationally, are identified as barriers to YADD exercising their privacy rights. The main factors shown to act as barriers can be categorized as relating to presentation of policy, gaps in legislation, and rise of corporate interests. Poorly written policy that is unclear (Dyke, et al., 2016; Keith, 2004; Law Commission of Ontario (LCO) 2017a; Mills et al., 2003; Rule, 2004; Wappel, 2007), and a lack of integration of technology into policy (Mills, et al., 2003) were cited often. The lack of international agreements and compliance with provincial and federal legislation in Canada, including respect for local norms around disability and privacy rights, was stated by several authors as problematic (Dyke, et al., 2016; Geist, 2016; Mills, et al., 2003; Siegel et al., 2009). Increasing levels of corporate lobbying interests and associated pressure to lower standards (Geist, 2016; Peekhaus, 2008) and exploitation of personal information (Peekhaus, 2008; Rule, 2004) were also cited as barriers.

On the other hand, facilitators of information privacy rights were also supported in the articles, primarily, the need for penalties to enforce accountability (Bearwood, 2015; Desai, 2013; Dyke, et al., 2016; Geist, 2016; Joffe, 2010; Keith, 2004; LCO, 2017a; Wedge, 2014), but also increased acknowledgement and respect for disability (Canadian Association of Community Living (CACL), 2011; Community Living Huntsville, 2012; Central East Local Health Integrated Network (LHIN), 2009; Repetto et al., 2008), community, socioeconomic, and technology support (CACL, 2011; Joffe, 2010; LCO, 2017b), and higher standards for security of personal information (Community Living Huntsville, 2012; Peekhaus, 2008).

### Exo Level: YADD and Community Interactions

At the exo level for adults with developmental disabilities, an interesting pattern of barriers and facilitators was evident consistently across articles. This related to access issues based in the interactions between YADD and their community and service providers. Barriers included insufficient knowledge leading to limited use of the existing legislation (Keith, 2004; Mental Health Commission of Canada (MHCC), 2015; Mills, et al., 2003; Repetto, et al., 2008; Rule, 2004; Simcoe York Dual Diagnosis Education Committee (SYDDEC), 2015; Wellington North, 2015), fear of reprisal for making a complaint (Joffe, 2010), lack of transparency regarding online social media platforms (Siegel et al., 2009; Rule, 2004), and use of overly complex language and presentation of rules (Siegel et al., 2009; Yalon-Chamovitz, 2009). Most noteworthy was the observation of a failure among organizations to provide appropriate accommodations to better support consent for collection and use of personal information and advocacy (Dyke et al., 2016; Joffe, 2010; Lafky & Horan, 2011; Larivie’re-Bastien & Racine, 2011; Mills et al., 2003; Wolbring, & Leopatra, 2013; Yalon-Chamovitz, 2009).

Facilitators identified at the exo level included increasing the use of advocacy supports by YADD and family caregivers (Joffe, 2010; Lafky & Horan, 2011; Mills et al., 2003), provision of technology for YADD to facilitate communication and comprehension (CACL, 2011; Larivie’re-Bastien & Racine, 2011), and most importantly, the inclusion of family members to support decision-making (Chan, & O’Brien, 2011; Desai, 2013; LCO, 2017a, b; MHCC, 2015; Peekhaus, 2008). Finally, the addition of specific instructions, in professional practice and organizational guidelines, on how to support young adults with developmental disabilities to exercise their privacy rights was cited in all articles discussing interactions with YADD in the community (Kitchener Downtown Community Health Centre, 2012; Law Commission of Ontario, 2017a, b; Legislative Assembly of Ontario, 2008; Repetto et al., 2008; Wellington North, 2015).

### Meso Level: Organization-Based Factors

Factors related to organizations and their processes, specifically to staff relations that act either as barriers or facilitators of access and utilization of privacy of information rights, were identified at the meso level. Barriers included a persistent lack of willingness on the part of organizations to share their information databases in order to ease the stress on YADD and their families (Clement, & Obar, 2016; Desai, 2013; Dyke et al., 2016; Gagnon et al., 2016; Larivie’re-Bastien, & Racine, 2011; Peekhaus, 2008; Mills, et al., 2003; Ministry of Health and Long-Term care (MHLTC), 2006; Lacobucci, 2014; Urowitz et al., 2008) and lack of commitment to use of technological advances to protect information privacy rights (Mills, et al., 2003; Siegel et al., 2009; Urowitz et al., 2008). Throughout the review, YADD needs were found to be unrepresented in organizational processes (Austin, 2006; CACL, 2011; Clement, & Obar, 2016; Siegel et al., 2009).

Identified solutions that acted as facilitators at the meso level focused on the provision of greater levels of experience, training, and practice opportunities for staff to better facilitate privacy rights for YADD (Dyke, et al., 2016; Gagnon et al., 2016; Lafky & Horan, 2011; Legislative Assembly of Ontario, 2008; Wellington North, 2015; Yalon-Chamovitz, 2009). As well, implementation of assessment of YADD needs for electronic health records and patient portals (Dyke et al., 2016; Urowitz et al., 2008) and enforcement of compliance among organizations in providing service that is consistent with existing privacy legislation and legislation governing the provision of individualized accommodations particularly for communication and comprehension (Austin, 2006; CACL, 2011; Dyke et al., 2016; Geist, 2016; Ontario Law, 2008).

### Micro Level: Individual Disability-Related Factors

A few characteristics that stem from the individual’s disability were identified as barriers at the micro level. However, it is important to note that existing legislation already provides direction to avoid these barriers but is often overlooked due to lack of compliance with privacy legislation and community-based care (Beardwood, 2015; Davidson et al., 2016; Dyke et al., 2016; Geist, 2016; Joffe, 2010;). For instance, the failure to provide individualized accommodations YADD need to exercise their rights and receive support for their decision-making due to low literacy as well as communication, comprehension, and visual difficulties surfaced consistently through this review (CACL, 2011; Joffe, 2010; Larivie’re-Bastien, & Racine, 2011; Repetto et al., 2008; Yalon-Chamovitz, 2009). Similarly, a lack of support to better prepare YADD during this transition period was identified as a major vehicle for YADD to become more empowered in exercising their information privacy rights (Davidson et al., 2016; Joffe, 2010; Larivie’re-Bastien, & Racine, 2011; Repetto et al., 2008). Finally, socioeconomic issues were cited as barriers to utilization of privacy rights, in that fees make it difficult for YADD and their families to make corrections to their personal information files (CACL, 2011; Laverie-Bastien, & Racine, 2011; Urowitz et al., 2008).

To better facilitate access and utilization of privacy rights for YADD, provision of support in two main areas were identified: (1) provision of training to improve computer literacy and self-advocacy skills specific to information privacy rights (CACL, 2011; Joffe, 2010; Repetto et al., 2008; Urowitz et al., 2008), and (2) provision of supported decision-making through greater engagement of family members in the consent process and utilization of privacy rights (CACL, 2011; Central East LHIN, 2009; Chan & O’Brien, 2011; Davidson et al., 2016; Lafky & Horan, 2011; LCO, 2017a, b; Joffe, 2010; Repetto et al., 2008).

### Recommendations

The findings and discussions of these articles show that the gap between the existing legislation and access to, or utilization of, information privacy rights among YADD and their families results from three factors: (1) a lack of knowledge among YADD and their family supporters (Gagnon et al., 2016; Joffe, 2010; Lacobucci, 2014; Lafky & Horan, 2011; Repetto et al., 2008; Yalon-Chamovitz, 2009), (2) a tendency of organizations to interpret or adhere to privacy laws inconsistently (Clement & Obar, 2016; Desai, 2013; Dyke et al., 2016; Geist, 2016; Peekhaus, 2008; Wolbring & Leopatra, 2013; Urowitz et al., 2008), and (3) a lack of access to the tools needed to support YADD exercising their information privacy rights (Davidson et al., 2016; Joffe, 2010; Larivie’re-Bastien & Racine, 2011; Mills et al., 2003; Repetto et al., 2008; Skouge et al., 2007; Urowitz, et al., 2008; Yalon-Chamovitz, 2009).

In connection with the findings reviewed, we recommend the following three areas that need to be addressed by privacy commissioners and organizations supporting young adults with developmental disabilities. First, and foremost, awareness, knowledge, and skills need to be raised among organizational staff, families, and YADD. In this way, self-advocacy will occur more frequently, and families will be included in the YADD’s core support and privacy rights education in their high school curriculum. Second, promotion of information privacy legislation through education for organizational staff is required. In particular, implementation of manuals with specific guidelines instructing staff with strategies to use the privacy legislation is suggested. Finally, targeted translation of existing legislation that will allow more YADD and their families to exercise their privacy of information rights is recommended.

## Limitations

This review has some limitations. Our literature search was limited to articles focusing on privacy legislation and developmental disabilities, and this search strategy may have contributed to excessively narrow range of articles included. Several exceptions for inclusion were established to capture relevant articles and to make it possible to broaden the capture of relevant barriers and facilitators also relevant to YADD. The articles differed not only in their objectives and methodology or type of article, but also showed great heterogeneity in approach to considering the issue of privacy of information rights. While quality was consistently high (such as representative samples, standardized tools, in depth policy discussion), the total number of empirical studies was very few (*n* = 6). It is important that future studies examine public understanding of privacy rights and pathways to accessing tools to exercise privacy rights. Finally, the presentation of our findings in Fig. 2 (Conceptual Framework: Determinants of Privacy of Personal Information Rights for YADD) presents a systems perspective. Our interpretation of our findings is influenced by this perspective.

## Conclusion

In this scoping review, 24 peer-reviewed articles and 23 articles from the gray literature were identified which examined the barriers and facilitators for access and utilization of information privacy rights in relation to the needs of young adults with developmental disabilities and their families. A basic requirement in protecting individual privacy rights is informing people of their rights so they know when a violation has occurred and how it can be remedied. This becomes a challenge to organizations trying to support families and their YADD as communication and information sharing can be complicated, often requiring accommodations or supports for the decision-making. The implications of the challenges typically experienced by YADD are tremendous, especially for providing support for provision of consent and decision-making to ensure ensuring confidentiality and autonomy throughout the protection of personal information privacy (Joffe, 2010). Although some parallels can be made with elderly citizens or persons with physical disabilities, important and distinct differences exist for YADD whose chronological age indicate adult, but who may not have sufficient decision-making capacity, and, more often than not, may be restricted as a result of their mode of communication or literacy level. By the time the potential privacy breach is voiced, or even recognized, young adults with developmental disabilities may have already lost some of their privacy rights in the process. For this reason, and because existing privacy legislation exists, the present review suggests that increasing awareness, education, and knowledge translation tools for all involved, especially YADD, family caregivers, and service providers, will help to combat the disadvantage in exercising information privacy rights by young adults with developmental disabilities.
